# Effects of anserine on oxidative stress and on cell barrier integrity in methylmalonic aciduria

**DOI:** 10.1038/s41598-025-20600-x

**Published:** 2025-09-25

**Authors:** Felix Köpfer, Maria Bartosova Medvid, Thomas Fleming, Tilman Pfeffer, Jürgen Günther Okun, Stefan Kölker, Georg Friedrich Hoffmann, Claus Peter Schmitt, Marina Morath, Verena Peters

**Affiliations:** 1 Division of Pediatric Neurology and Metabolic Medicine, Department of Pediatrics I, Medical Faculty Heidelberg, Center for Pediatric and Adolescent Medicine, Heidelberg University, 69120 Heidelberg, Germany; 2 Medical Faculty Heidelberg, Internal Medicine I and Clinical Chemistry, University Hospital Heidelberg, Heidelberg University, 69120 Heidelberg, Germany

**Keywords:** Human immortalized kidney tubule cell, Methylmalonic aciduria, Oxidative stress, Anserine, Metabolic cell stress, Diseases, Medical research, Nephrology, Pathogenesis

## Abstract

Kidney damage in individuals with methylmalonyl-CoA mutase deficiency (*mut*^0^) results from metabolic and oxidative stress, and disrupted mitochondrial homeostasis. Anserine, known for its antioxidant properties and protective effect on cell barrier integrity of endothelial and epithelial kidney and vascular cells, may offer therapeutic potential for chronic disorders. This study explored anserine’s effects on immortalized kidney tubular epithelial cells (iKTEC) from *mut*^0^ patients. Compared to healthy controls, iKTEC from *mut*^0^ patients showed reduced cell viability, antioxidant capacity, oxygen consumption, and ATP production. Expression of the tight junction scaffolding protein zonula occludens-1 (ZO-1) was increased in *mut*^0^ cells compared to control while transepithelial resistance (TER), and dextran transport (10 kDa) remained unchanged. Anserine treatment restored antioxidant capacity and normalized ZO-1 expression but had no effect on TER or dextran transport. Additionally, cell viability, mitochondrial respiration, and ATP production were unaffected by anserine. Metabolic stress induced by high protein load or disease-associated branched-chain amino acids did not worsen mitochondrial dysfunction or epithelial integrity, and anserine exposure showed no further effects. In conclusion, anserine showed promise in restoring antioxidant capacity in iKTEC from *mut*^0^ patients, highlighting its potential as a therapeutic agent to mitigate ROS, although its effects on mitochondrial function and epithelial integrity warrant further investigation.

**Supplementary Information:**

The online version contains supplementary material available at 10.1038/s41598-025-20600-x.

## Introduction

Methylmalonic aciduria (MMA) is the biochemical hallmark of a genetically defined group of rare disorders characterized by impaired activity of methylmalonyl-CoA mutase (MMUT), a specific enzymatic step in the anaplerotic propionate metabolism which channels converging catabolic pathways of the amino acids isoleucine, methionine, threonine, and valine, odd-chain fatty acids, and intestinally derived propionate, or by impaired metabolism of its cofactor adenosylcobalamin. Inherited deficiency of *MMUT* may be complete (*mut*^0^; OMIM 251,000) or partial (*mut*^−^; OMIM 251,000). The extent and the severity of clinical symptoms is related to the underlying defect. While *mut*^0^ forms are usually associated with a severe acute life-threatening neonatal metabolic crisis and the development of widespread late organ complications although therapeutic intervention, the clinical picture in *mut*^-^ forms is much more variable due to the different MMUT residual activity resulting in a wide spectrum of mild to severe disease^1,2^. Treatment strategy consists of dietary restriction of precursor amino acids, supplementation of carnitine, avoiding catabolism, parenteral vitamin B12 supplementation and in the long-term liver and/or kidney transplantation^3^. In early childhood, neurological abnormalities such as developmental delay, cognitive deficits, seizures or movement disorders are most common. Later, depending on the mutation, a clinical picture of irreversible secondary multi-organ damage develops. The majority of *mut*^0^ patients, and less frequently *mut*^-^ patients, develop secondary renal damage despite appropriate therapeutic measures, which usually leads to terminal renal failure in adolescence^2^. Renal damage in MMA is complex and multifactorial, likely triggered by prolonged exposure to high toxic intermediates, such as propionyl-CoA, 2-methycitrate and methylmalonic acid leading to oxidative stress and mitochondrial dysfunction^4^; partially due to impaired electron transport chain function^5–9^. This dysfunction is exacerbated by the accumulation of toxic metabolites during catabolism or by protein-load of the deficient pathway resulting in a serious disturbance of complex mitochondrial network homeostasis like e.g. quality control or dynamics^4,9–13^.

The therapeutic potential of carnosine and anserine, two histidine-containing dipeptides with protective properties, has been demonstrated in various diseases^14–19^. Their mode of action can be attributed to various properties, such as a high antioxidant potential by activating the intracellular defence system during oxidative stress, protective actions on epithelial and endothelial barrier disintegration and dysfunction, carbonyl-quenching and inducing H_2_S formation^20–24^. Carnosine preserves membrane potential and improves mitochondrial defence mechanisms^25–27^, studies on the effect of anserine on mitochondrial function are lacking. The effects of anserine on the epithelial barrier, crucial in the development of kidney disease^28,29^ has been recently demonstrated^24^. Further, previous results have demonstrated that anserine reduced vascular permeability in the kidneys of mice^18^ and carnosine supplementation has been discussed in patients with peripheral vascular disease^30^. However, it remains unclear whether these changes are caused directly by the dipeptides or indirectly by an increase in antioxidant capacity.

Since anserine has a higher antioxidant capacity and a higher protective effect on cell barrier integrity, compared to carnosine, we investigated the potential of anserine on renal cells using well characterized iKTEC from *mut*^0^ patients^4,9,11,13^. The human proximal and distal tubule has a central role in water, ion and small nutrient transport and plays a critical role in the progression of kidney disease^31^. We examined the antioxidant response and its effect on mitochondrial activity and investigated the epithelial barrier function by measuring transepithelial resistance (TER) and the tight junction protein Zonula occludens (ZO-1) being a key protein that maintains the integrity and assembly of these junctions and for osmotic pressure regulation of cell volume^32,33^. The cells were additionally exposed to various stress conditions by well-described metabolic stressors, such as high protein concentration, addition of disease-associated amino acids (isoleucine/valine), propionate, an intermediate precursor metabolite of deficient metabolic pathway, and Fenton-induced oxidative stress^4,9^ to intensify the effect.

## Material and methods

### Primer, antibodies and chemicals

MMUT Primer (For: CGAATTGCCAGGAACACACA; Rev: TTCTAGCTTGTCTTCGGGCA), p53 Primer (For: GCG AGCACTGCCCAACAACA; Rev: GGAGACATCGTCTGGGGTGT), HO-1 Primer (For: AAGACTGCGTTCCTGCTCAACRev: AAAGCCCTACAGCAACTGTCG), y-GCS Primer (For: GGCACAAGGACGTTCTCAAG; Rev: CTGTCCTGGTGTCCCTTCAA), KEAP-1 Primer (For: GCTGTCCTCAATCGTCTCCT; Rev: TCCACGTCTCTGTTTCCACA). All primers purchased from Thermo Fisher Scientific, Waltham, USA. MMUT polyclonal antibody (Proteintech Group, Rosemont, USA), ZO-1 Monoclonal Antibody, Alexa Fluor® 555 (Thermo Fisher Scientific, Waltham, USA), β-Actin (C4) HRP mouse monoclonal (Santa Cruz Biotechnology, Dallas, USA). Dextran 9–11 kDa unlabeled and 10 kDa fluorescein isothiocyanate (FITC) labelled dextran (both obtainend from Sigma Aldrich, Darmstadt, Germany), DreamTaq Green PCR Master Mix (2x), Random Hexamers, Superscript IV Reverse Transkriptase (all obtained from Thermo Fisher Scientific, Waltham, USA). Seahorse XF Calibrant Solution 500 mL (Agilent, Santa Clara, USA). SYBR Green JumpStart Taq Ready Mix (Sigma-Aldrich, Darmstadt, Germany), peqGOLD total RNA KIT (VWR, Darmstadt, Germany), ATP / ADP Ratio Assay Kit (Sigma-Aldrich, Darmstadt, Germany) OxiSelectTM Oxygen Radical Antioxidant Capacity (ORAC) Activity Assay (Cell Biolabs, San Diego, USA).

### Cell culture

The human cells used were immortalized kidney tubule epithelial cells (iKTEC) obtained from the urine of healthy controls and from three *mut*^0^ patients (Table 1)^11^. The iKTEC which were used were previously characterized in detail^11,13^.

**Table 1:** Biochemical and genetic characterization of included patient cell lines. Data expressed as mean ± SEM (n = 3). Enzymatic activity of healthy individuals was between 3403 ± 136 and 7406 ± 336 pmol/min/mg. Table modified from^9,11^. mut^0^_1 and mut^0^_2 correspond to mut^0^_ 2 and mut^0^_ 3 in Ref.^9^.

iKTEC line	Age	Sex	Mutation		Enzymatic activity (pmol/min/mg)
			Nucleotide change	Amino acid change	
*mut0_1*	22	w	c.862 T > C (exon 4)	p.S288P; p.S288P	17 ± 10
*mut0_2*	23	m	c.982C > T (exon 4)	p.L328F; p.L328F	38 ± 25
*mut0_3*	16	m	c.826 T > C (exon 4); c.1157A > G (exon 6),	p.S288P; p.H386R	24 ± 15

Cells between passage 3 and 15 were grown in incubators with 37 °C and 5% CO2. Gibco™ DMEM GlutaMaxx medium with a high glucose content (4.5 g/l) with 10% fetal bovine serum (FBS) and 1% penicillin/streptomycin was used for cell cultivation and for the simulation of normal conditions (NM) in experimental setting. Stress was induced by high protein load (HP) with 25% FBS, high concentrations of branched chain amino acids (3 mM isoleucine and 1 mM valine; IV) and 5 mM propionic acid (Prop) and oxidative stress was induced by addition of 3 nM Fenton’s reagent (Fen). Stress conditions were maintained under normal incubator settings (37 °C and 5% CO2) for different time intervals (48 and 96 h; 7 days) after the cell layers had formed for 12 h. The basic medium for all stress conditions consisted of DMEM GlutaMaxx with low glucose content (1 g/l). To test anserine as a protective agent, it was added when stress conditions were induced. A concentration of 2 mM anserine was used in all experiments. Cell lines were routinely screened for mycoplasma contamination using a PCR-based assay^34^. In case of contamination, cells were treated with 25 µg/ml plasmocin (InvivoGen, USA) for 2 weeks^35^. Cells were only used after they were tested negative (this concerned cell line 2) .

### A patient consent statement/details of ethics approval

All individuals gave their informed consent.

The study was carried out following the criteria of the Helsinki Declaration of 1975, as revised in 2000.

The generation of the cell lines was approved by the ethical committee of Heidelberg University (ethical vote: (S-436/2016).

### Organic acid analysis

Methylmalonic acid was measured in plasma and urine of the investigated mut^0^ patients by isotope dilution gas chromatography–mass spectrometry as previously described in an accredited laboratory^2,36^.

### MMUT activity

MMUT was assayed in crude cell homogenates in the presence (total *MMUT* activity) and in the absence (holo-*MMUT*) of the cofactor adenosylcobalamin as described before^13^.

### Cell viability

The MTT assay was used to determine cell viability. The cells were seeded at a density of 10,000 per well and allowed to grow overnight in NM. The next day, the medium was removed, and incubation time was started with the different stress conditions. For all models, the cells were stressed for 48 and 96 h. After incubation, 50 µl MTT (3-(4,5-dimethylthiazol-2-yl)-2,5-diphenyltetrazolium bromide) reagent was added. After 4 h of incubation, cell lysis was then performed using dimethyl sulfoxide (DMSO). The quantification of converted MTT was conducted photometrically.

### Western blot

Protein electrophoresis was performed to separate the size of protein mixtures; protein lysates were mixed with dH_2_0 and 6 × loading dye, denatured and loaded onto a polyacrylamide gel. The gel electrophoresis allowed the proteins to be separated according to their size. After electrophoresis the proteins were transferred to a nitrocellulose membrane using a semi-dry blotting technique. The primary antibody of the target protein was allowed to bind overnight, the secondary antibody was then added for one hour. The analysis was performed on a Fusion SL4 (Peqlab). For semi-quantitative analysis, densitometry was performed using ImageJ software. A β-actin antibody was selected as a loading control and the densitometric values were normalized to the β-actin density and quantified.

### Real time PCR

Gene expression analysis was also performed at RNA level. RNA extraction was performed using the peqGold Total RNA Kit (VWR). For the next step, cDNA synthesis, 1000 ng RNA per sample was mixed with nuclease-free water dNTP mix and random hexamers primer. cDNA synthesis was performed via standard thermo cycler protocol. The cDNA samples were mixed with SYBR Green Mix and each of the sense and antisense primer. The subsequent qRT-PCR reaction was performed according to a standard procedure scheme using β-actin as reference gene.

### Transepithelial resistance

To create an in vitro model of an epithelial monolayer, the iKTECs were seeded in transwell filters. An apical and basal compartment could be distinguished, and the electrical voltage could be measured with a volt-ohm meter between the different sections. The electrical resistance (in Ω/cm^2^) of each cell monolayer was calculated using the background TER and the growth area of the filter.

#### Paracellular protein transport

Paracellular permeability of epithelial monolayers was determined by measuring the flux of 10 kDa fluorescein isothiocyanate (FITC)-labeled dextran from the apical to the basolateral compartment of a transwell® insert. An equimolar amount of unlabeled dextran was added to the basolateral compartment. At the end of the assay, transported samples were collected from the basal compartment and fluorescence was measured using the Tecan Spark Plate Reader.

#### Immunohistochemical staining

Immunofluorescent staining of proteins and organelles in cultured cells was also carried out in the permeable membrane filters. First the cells were fixed, then permeabilized before starting the incubation with primary and secondary antibody. Tight junctions were visualized with ZO-1 555 antibody and DAPI staining was used to visualize cell nuclei. The membrane was detached from the filters and fixed to a glass slide. After 24 h, images of the immunostained cells were captured with an Imaging Machine (ACQUIFER). The images were quantified and analyzed using the ImageJ 1.53 k (Wayne Rasband, open source) software based on the number of pixels in specific areas.

#### Determination of oxidative stress

The ROS caused by reactive oxygen species was measured intracellularly using the OxiSelect™ Activity Assay Kit (ORAC). The cells were incubated for a short (48 h) and long (7 d) interval in normal medium and the different stress medium with and without the addition of anserine. The time-dependent fluorescence measurement was performed with the SPARK® Multimode Microplate Reader. The antioxidant effect of the vitamin E analog Trolox™ was used as a standard curve and used in the calculation.

#### Mitochondrial energy production

Mitochondrial respiration was measured using the Seahorse Bioanalyzer (Aligent;^37^). Cells of interest were harvested, collected, counted and reseeded into XF96cell culture plate (Agilent) at density of 30,000 cell per well and allowed to adhere overnight. The next day the media was changed to the Agilent Seahorse XF Assay Medium (Agilent) supplemented with 10 mM glucose, 2 mM glutamine, 1 mM sodium pyruvate and incubated for 1 h prior to assay in a non-CO_2_ incubator at 37 °C. Injections of oligomycin (2 µM final), FCCP (1 μM final), a combination of rotenone and antimycin-A (0.5 μM final each) were diluted in the Agilent Seahorse XF Assay Medium and loaded onto ports A, B and C respectively. The bioanalyzer was calibrated and the assay was performed using Mito Stress Test protocol as suggested by the manufacturer (Agilent Seahorse Bioscience®). The assay was run in one plate with 4 technical replicates per condition. Seahorse Wave software (version 2.6.1.53; https://www.agilent.com/en/product/cell-analysis/real-time-cell-metabolic-analysis/xf-software/seahorse-wave-desktop-software-740897) was used to analyze metabolic data generated from both assays. The data from each assay was normalized to the total protein content in each well, as measured by the Bradford assay^38^. Following addition of lysis buffer (100 mM sodium phosphate (pH7.4) + 0.1% Triton X100; 50 µl per well) and two cycles of freeze-thawing.

#### Anserine treatment

2 mM anserine were added to the cell medium. The concentration was based on the previous studies with anserine treatment of human tubular cells. The duration of treatment varied from one to seven days; a further description can be found in the respective figure legend. In previous studies, we demonstrated that human tubular cells can tolerate high doses of anserine, with concentrations ranging from 1 to 70 mM, antioxidative response was visible within a range of 1–10 mM Anserine^23,24,39^.

#### Statistical analysis

All results are presented as mean ± SD for continuous variables, but as median for ordinal scaled data. For all cell studies, we used 3 patient-derived cell lines (3 biological replicates), each tested in at least 3 experimental replicates (quantity of experiments of the same sample as shown in the figures). Each experimental replicate was based on 5–9 technical replicates (quantity of measurements in the same experimental condition). A significance level of 5% was set for all analyses. For comparisons between only 2 cohorts, a two-tailed t-test was used. To show differences in three or more different groups, a two-way ANOVA with Šídák’s multiple test was used. In all figures statistical analysis of data was performed between control cells and *mut*^0^ cells within the same normal or stress condition and also between the same cell lines with or without the addition of anserine. When normalizing the experiment results, this was usually done to the values of the control cells under normal conditions (NM) in the respective experiment. The measured values were presented as diagrams and plots using the computer application GraphPad Prism software (version 10.2, San Diego, USA, available at: https://www.graphpad.com).

## Results

### Characterization of cells

**Fig. 1:**
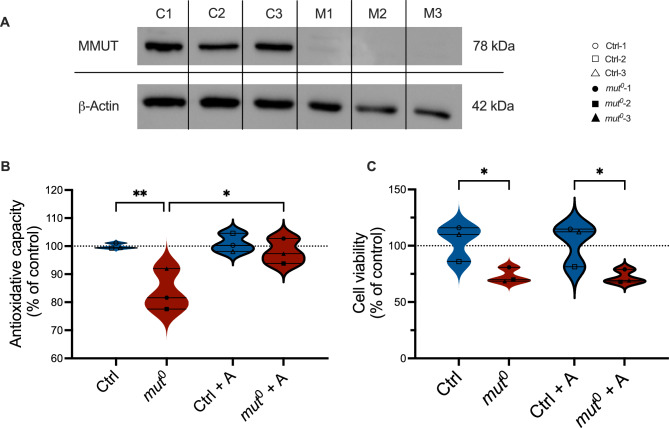
Effect of anserine on antioxidative capacity and cell viability in *mut*^0^ and control cells. (**A**) The presence of the genetic defect in immortalized kidney tubule epithelial cells (iKTEC) from three patients with clinically and molecular biologically confirmed loss of activity of methylmalonyl-CoA mutase (MMUT) was confirmed at protein level (by Western blot) before performing the experiments. C1-3 = Control patients; M1-3 = patients with *mut*^0^ defect. The figure is displayed as cropped version of the performed western blot, full-length gel is included in the supplementary information (Suppl. Fig. 1). (**B**) Antioxidative capacity, measured by ORAC, was reduced in iKTEC from 3 patients with methylmalonyl-CoA mutase deficiency (*mut*^0^) compared to immortalized cells from healthy individuals (Ctrl). Incubation with anserine (A, 2 mM) for 7 days increased the antioxidative capacity in cells from *mut*^0^ patients. (**C**) Cell viability (MTT assay) of iKTEC from patients was reduced in iKTEC from 3 *mut*^0^ patients. Incubation with anserine (2 mM) for 96 h had no influence on cell viability in cells from patients and controls. Statistical analysis was performed using a simple t-test for A and ordinary two-way ANOVA with Šídák’s multiple comparisons test for B and C.* = *p* < 0.05; ** = *p* < 0.01; **** = *p* < 0.0001. There is no significance between the groups, except for the ones indicated in the graphs. Antioxidant capacity and viability are shown as percentages relative to control cells in normal medium (dashed line = 100%). Symbols/lines in the violin plots represent the mean value for one patient analyzed in three independent experiments, each performed with 5–9 technical replicates.

Patients with *mut*^0^ defect have a genetically determined deficiency of the enzyme MMUT. The iKTEC originate from three genetically confirmed *mut*^0^ patients (Table 1). The deficiency of the enzyme in our cells could be confirmed at protein level (Fig. 1A) and enzymatically (Table 1). The long-exposure Western blot (WB) revealed residual protein concentrations that do not appear to correlate with protein function (Suppl. Fig. 1), as also indicated by the absence of measurable enzymatic activity (residual MMUT activity of approximately 1–2%, consistent with previous reports^4,8,9^ (Table 1)), accompanied by high concentrations of methylmalonic acid (above 3000 (*mut*^0^_1), 4000 (*mut*^0^_2) and 5500 (*mut*^0^_3) µmol/L in plasma and above 5000 (*mut*^0^_1), 4000 (*mut*^0^_2 ) and 5500 (*mut*^0^_3) mmol/mol creatinine in urine), in contrast to the reference ranges observed in healthy individuals (0–0.26 µmol/L in plasma and 0–10 mmol/mol creatinine in urine).

### Effect of anserine on antioxidant capacity

The antioxidative capacity was reduced by 20% in the iKTEC from 3 *mut*^0^ patients compared to healthy controls. Incubation with anserine (2 mM) significantly restored the reduced antioxidant capacity in iKTEC from *mut*^0^ patients to near-normal levels after incubation of 7 days (Fig. 1B). However, the mRNA expression of antioxidative stress response markers, including heat-shock protein heme oxygenase (HO-1), the stress sensing protein Kelch-like ECH-associated protein 1 (KEAP-1), key enzyme of the glutathione system gamma-glutamylcysteine synthetase (γ-GCS), and the tumor suppressor gene p53, showed no significant differences between patient and control cells after incubation of 96 h (Suppl. Fig. 2). Furthermore, stress induced by exposure to high-protein (HP) and branched-chain amino acid (IV) load did not affect antioxidative capacity or mRNA expression of the stress markers. Anserine was unable to increase the antioxidant capacity in the metabolically stressed cells (Suppl. Fig. 2).

### Effect of anserine on cell viability

The cell viability of the iKTEC from 3 *mut*^0^ patients was by 20 and 25% lower compared to control cells at 48 and 96 h, respectively (Fig. 1C; Suppl. Fig. 3). Anserine incubation (2 mM) had no impact on cell viability in either control and *mut*^0^ cells (Fig. 1C). To further elucidate viability differences, various stress models were employed to specifically stress the *mut*^0^ and control cells. Metabolic stress by adding high-protein (HP, 25% FCS), disease associated branched-chain amino acids (IV, 1 mM isoleucine, 3 mM valine), propionic acid (Prop, 5 mM), an intermediate precursor metabolite, or oxidative stress induced by Fenton (Fen, 3 nM) did not significantly enhance the difference between control and *mut*^0^ cells. Reduced viability by stress was within the same range for control and patient cells. Incubation with anserine did not improve cell viability under any of the conditions (Suppl. Fig. 3).

### Effect of anserine on mitochondrial energy production

**Fig. 2:**
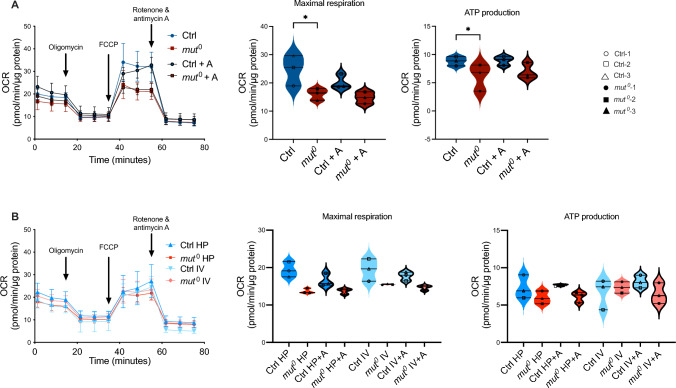
Effect of anserine on mitochondrial energy production measured by real-time respirometry in *mut*^0^ and control cells. (**A**) Mitochondrial oxygen consumption rate (OCR) was lower in immortalized proximal tubule epithelial cells (iKTEC) from *mut*^0^ patients compared to control cells. Basal respiration, ATP production (area under the curve after oligomycin injection) and maximal respiratory rate (area under the curve after FCCP injection) was lower in *mut*^0^ cells compared to control cells. Incubation with anserine (A, 2 mM for 24 h) had no effect on ATP production in *mut*^0^ cells compared to control cells (*p* = 0.143 and *p* = 0.140). (**B**) Metabolic stress by High Protein load (HP) or branched-chain amino acids (IV, 1 mM isoleucine, 3 mM valine) did not further reduce mitochondrial oxygen consumption rate (OCR) compared to non-stressed iKTEC cells from *mut*^0^ patients. Incubation with anserine (A, 2 mM for 24 h) had no effect on the OCR in stressed cells. Statistical analysis was performed by using two-way ANOVA with Šídák’s multiple comparison test. * = *p* < 0.05; ** = *p* < 0.01 compared to control cells. Symbols/lines in the violin plots represent the mean value for one patient analyzed in three independent experiments, each performed with 5–9 technical replicates. There is no significance between the groups, except for the ones indicated in the graphs.

In unstressed *mut*^0^ patient iKTEC, mitochondrial oxygen consumption rate (OCR) was notably lower compared to controls. Maximum respiration rate, calculated as the area under the curve between 40 and 60 min, exhibited reduced respiratory rates in *mut*^0^ cells. Co-incubation with anserine (2 mM) for 24 h did not alter OCR. Furthermore, ATP production, assessed between 15 and 35 min, was diminished in *mut*^0^ cells compared to controls (Fig. 2A). High protein- and branched-chain amino acid-induced stress reduced respiratory capacity in both control and *mut*^0^ cells. Co-incubation with anserine had no effect on maximal respiration and ATP production (Fig. 2B).

### Effect of anserine on epithelial cell barrier

**Fig. 3:**
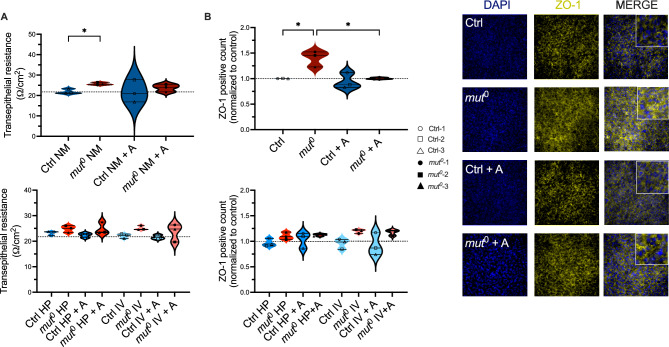
Effect of anserine on transepithelial resistance and ZO-1 localization and abundance. (**A**) Transepithelial resistance (TER), a measure of barrier integrity, was increased in *mut*^0^ cells compared to controls (Ctrl). Co-incubation with anserine (A, 2 mM) for 7 days had no effect on TER in cells from *mut*^0^ patients (upper panel). Metabolic stress by High Protein load (HP) or branched-chain amino acids (IV, 1 mM isoleucine, 3 mM valine) did further increase TER (lower panel). (**B**) Expression of the tight junction scaffolding protein zonula occludens-1 (ZO-1) was increased in *mut*^0^ cells compared to controls (Ctrl), expression could be normalized by addition of anserine for (A, 2 mM) in *mut*^0^ cells after an incubation period of 7 days. Metabolic stress by High Protein load (HP) or branched-chain amino acids (IV, 1 mM isoleucine, 3 mM valine) did not further increase ZO-1 expression. Analysis was performed after 5 days of growth. Grid line shows TER and ZO-1 levels of Ctrl in every graph. If not further explained, cells were treated with normal medium. Statistical analysis was performed by two-way ANOVA with Šídák’s multiple comparisons test. Symbols/lines in the violin plots represent the mean value for one patient analyzed in three independent experiments, each performed with 5–9 technical replicates. * = *p* < 0.05; ** = *p* < 0.01. There is no significance between the groups, except for the ones indicated in the graphs.

**Fig. 4:**
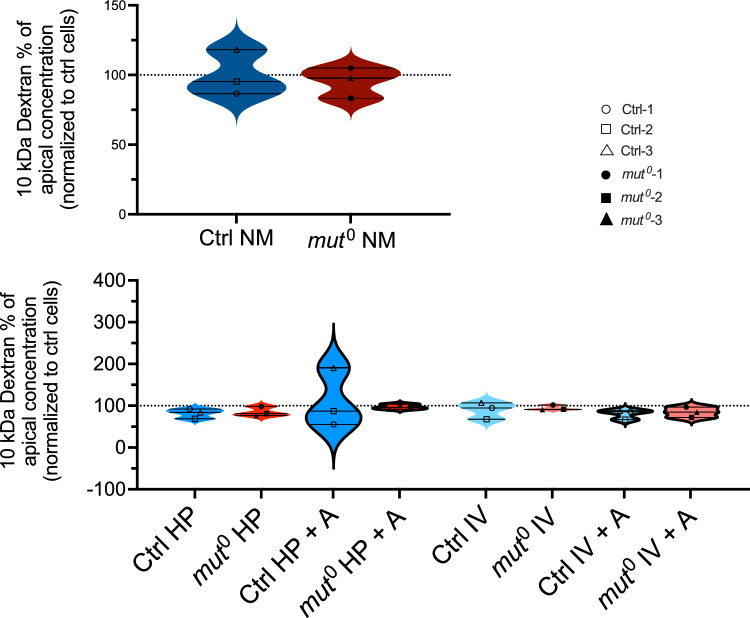
Effect of anserine on 10 kDa transport. Transport capacity for 10 kDa dextran was within the same range for *mut*^0^ and control cells (upper panel). Metabolic stress by High Protein load (HP) or branched-chain amino acids (IV, 1 mM isoleucine, 3 mM valine) had no additional effect on transport. Treatment with anserine for 7 days did not alter transport capacity. Dots in the violin plots represent the mean value for one patient analyzed in three independent experiments, each performed with 5–9 technical replicates. Statistical analysis was performed by two-way ANOVA with Šídák’s multiple comparisons test. There is no significance between the groups.

The transepithelial resistance (TER) was higher in *mut*^0^ cells compared to the control cells. Anserine could not normalize the increased TER in patient cells (Fig. 3A). Under stress conditions, TER tended to increase in *mut*^0^ cells under stress, while no effect of stress conditions on TER were observed in controls (Fig. 3A). Expression of the tight junction scaffolding protein zonula occludens-1 (ZO-1) was increased in *mut*^0^ cells compared to controls (Ctrl), expression could be normalized by addition of anserine for (A, 2 mM) in *mut*^0^ cells. Metabolic stress by High Protein load (HP) or branched-chain amino acids (IV, 1 mM isoleucine, 3 mM valine) did not further increase ZO-1 expression in *mut*^0^ cells. (Fig. 3B). However, anserine had no impact on small molecule transport properties. Protein transport of 10 kDa dextran was in the same range in patient cells and controls and remained unaffected by stress conditions (Fig. 4).

## Discussion

Despite various strategies, current treatment for MMA remains limited, and new therapeutic concepts, such as genomic technologies or disease-modifying therapies, are needed^40–42^. Since mitochondrial dysfunction in MMA, which is resistant to conventional therapy, is a major contributor to the secondary multi-organ damage in these patients and the formation of ROS form a vicious circle^43^, disrupting this loop through antioxidant treatment is a conceivable approach. There is already evidence of a repairing effect of mitochondrial function with antioxidants in the cell, transgenic mice and zebrafish models of MMA^12,13^. In addition, studies using the cell culture model presented here have shown that metabolic stressors like protein or precursor metabolites of the deficient pathway impair significantly mitochondrial antioxidant defense system in *mut*^0^ and also in propionic aciduria^4,9^.

Based on these findings, we now investigated the protective effect of the antioxidant dipeptide anserine on human renal epithelial cells from genetically, enzymatically and clinically confirmed MMA patients. The antioxidative effect of anserine on late diabetic kidney complications in diabetes has been demonstrated in several studies^18,21^. Further, MMAs often result in renal dysfunction. Since the barrier integrity of polarized cells is crucial for the physiological functions of the kidney, we investigated cell barrier integrity by measuring TER and the tight-junction associated protein ZO-1 in patient’s cells. Tight junctions together with their adaptor proteins ensure proper barrier function and control paracellular transport in epithelial and endothelial cells^28,44^.

However, our study demonstrated that treatment of *mut*^0^ cells with anserine increased antioxidant capacity of the cells but did not restore mitochondrial bioenergetics, failing to disrupt this reinforcing cycle. Additionally, metabolic stress did not further exacerbate cell viability, antioxidative capacity or mitochondrial bioenergetics in the *MMUT* deficient cells, at least not during the treatment period of several days, which suggests that this perturbation is already maximal in the human *mut*^0^-cell model. The stable cell viability under metabolic stress observed in this study aligns with findings from other studies^9^. However, no additional impairment of the antioxidant capacity, as evidenced by glutathione homeostasis, was detected here. This discrepancy may be attributed to differences in the methodologies used. Specifically, the ORAC assay quantifies total oxidative capacity, while the glutathione assay focuses on specific aspects of antioxidant function. In *mut*^0^ cells, mitochondrial bioenergetics were comprised, which, in contrast to other antioxidants such as Mito-Tempo^13^, could not be improved by anserine. Anserine has been shown to restore antioxidant capacity in *mut*^0^ cells. However, this effect is neutralised by metabolic stress, indicating the importance of a balanced metabolic situation in these patients. Metabolic stressors, such as a protein excess or catabolic periodes, exacerbate the vicious cycle of mitochondrial dysfunction, and may neutralise the potential reparative effects of disease-modifying substances.

Based on our previous findings that anserine can normalize carbonyl stress-induced damage and dysfunction of the epithelial and endothelial barriers, we further characterize key kidney tubule function in *mut*^0^ cells by measuring the general ion permeability and essential transport functions. TER was elevated in *mut*^0^ cells compared to cells from healthy individuals, indicating enhanced epithelial tightening. We further investigated its effects on endothelial resistance in cells derived from *mut*^0^ patients^24^. Contrary to our expectations, the patient cells did not show reduced barrier integrity. Previous studies have shown that indole-3-propionic acid, a branched chain amino acid metabolite, in a Caco-2/HT29 co-culture model resembling the human small intestinal epithelium, resulted in an increase in TER^45^. Whether the accumulation of disease-associated amino acids or propionate is responsible for increased TER in the renal *mut*^0^ cells needs further investigations. In Carnosinase 2-knock-out cells, increased TER was accompanied by impaired and altered ion and macromolecule transport via trans-and paracellular pathways^33^. However, treatment with anserine could not normalize TER, even though it was able to normalize the ZO-1, a tight-junction associated protein^46^, concentration. In kidney, the paracellular permeability decreases from the proximal tubule to the collecting duct, accompanied by increasing abundance of ZO-1 and varying expression levels of tight junction components^46^.

Interestingly, the various stress models had little influence on cell viability, mitochondrial activity or transepithelial integrity, in accordance with recently published data^9^. Of note, the iKTEC, albeit widely used, may only partially reflect metabolic functions, different results may be obtained with primary human kidney tubular epithelial cells in vitro, and in kidneys in vivo. These in vitro data also show only very short-term effects of anserine on the function of the cells, but the development of kidney dysfunction in the patients takes many years^47^.

In conclusion, our study demonstrated that anserine treatment increased antioxidant capacity in *mut*^0^ cells but did not normalized elevated transepithelial resistance, reduced cell viability or mitochondrial dysfunction. These findings suggest that the pathophysiological alterations in *mut*^0^ are not primarily ameliorated by protection against oxidative stress. However, further studies would be needed to show whether antioxidant components such as anserine could slow down the pathophysiological damage.

## Supplementary Information

Supplementary Information.

## Data Availability

All data generated or analysed during this study are included in this published article (and its Supplementary Information files). Individuals’ data are not publicly available due to the data protection law.
