# Supplementary material for: Effects of anserine on oxidative stress and on cell barrier integrity in methylmalonic aciduria
Source: Sci Rep. 2025 Sep 25;15:32933. doi: 10.1038/s41598-025-20600-x (PMC12464228; doi:10.1038/s41598-025-20600-x)
Supplement: Supplementary file 1 — Supplementary Information. [file 41598_2025_20600_MOESM1_ESM.pdf]

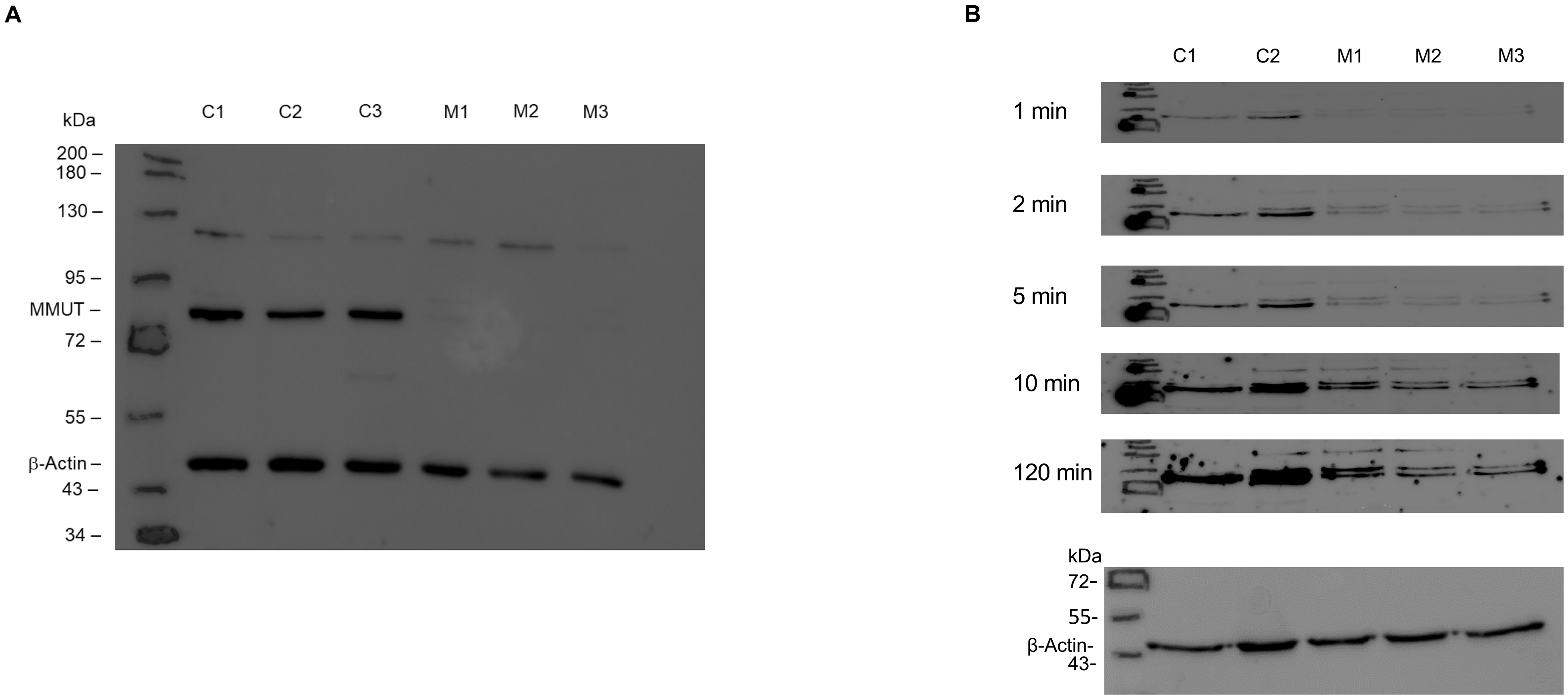

Suppl. Fig. 1: Additional western blots

A) Full western blot of methylmalonyl-CoA mutase (MMUT) in patients with *mut*^{0} defect (M1-M3) and in healthy controls (C1-C3). β-Actin was used as loading control. Exposure time was 50 seconds.

B) Long exposure western blot of methylmalonyl-CoA mutase (MMUT) in patients with *mut*^{0} defect (M1-M3) and in healthy controls (C1-C2): Exposure time varied between 1 and 120 minutes. β-Actin was used as loading control (exposure time 10 seconds). Normalizing the MMUT bands to β-Actin showed residual protein concentrations at 120 minutes exposure time 31 (for M1), 23 (M2) and 29 (M3) % for all compared to healthy controls

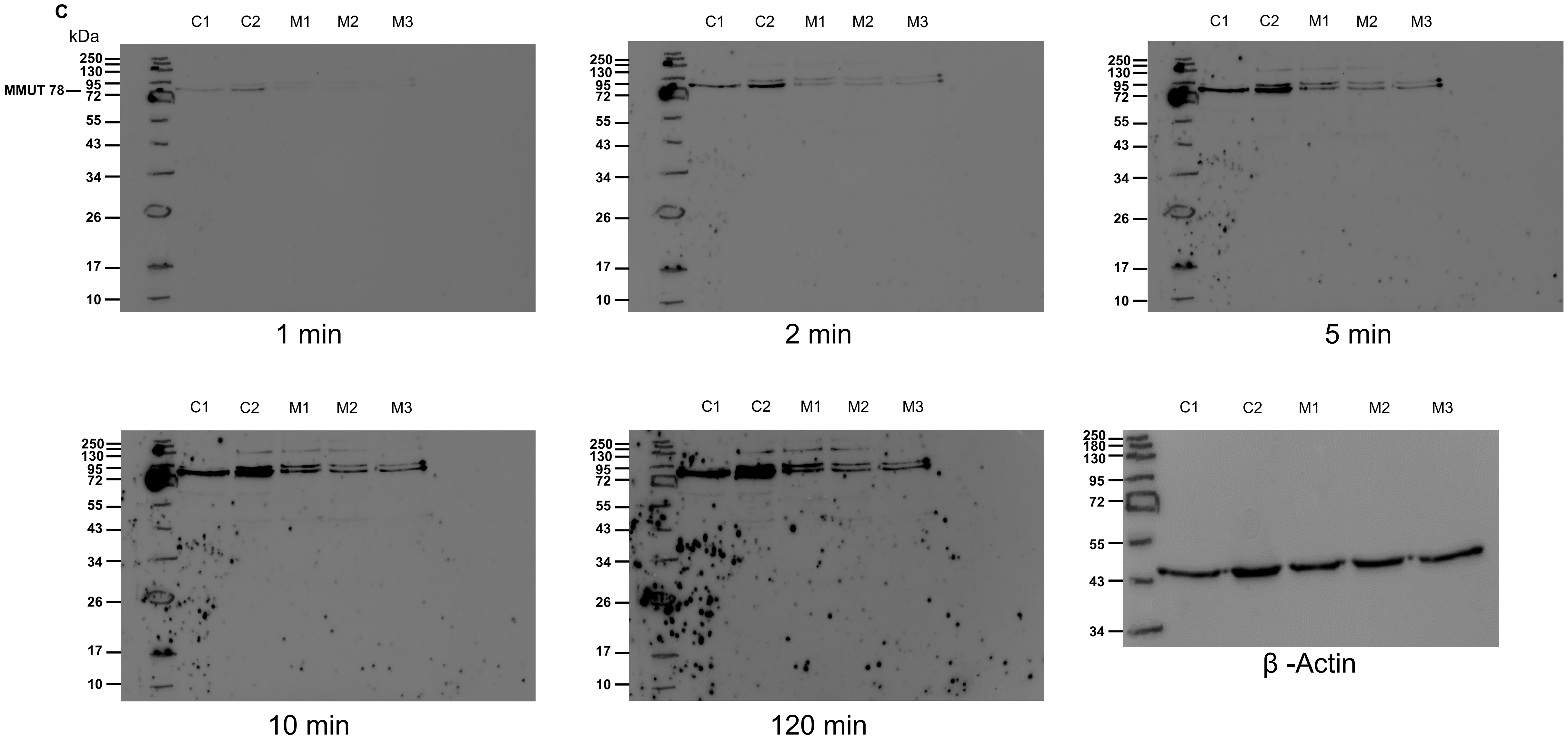

Suppl. Fig. 1: Additional western blots
C) Full western blot of the long exposure western blot (Suppl. Fig 1B)

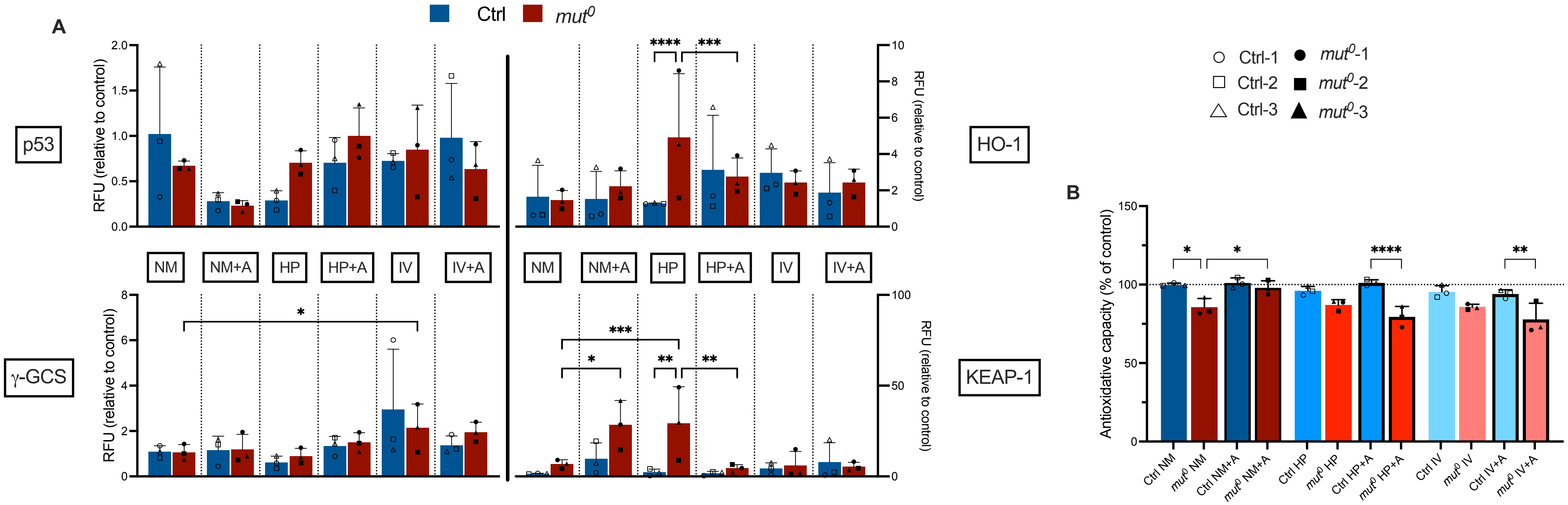

Suppl Fig. 2: Gene expression analysis of oxidative stress markers und stress conditions

A) Antioxidative response was measured by mRNA expression of p53, Heme oxygenase (HO-1), Kelch-like ECH-associated protein 1 (KEAP-1) and γ-glutamylcysteine synthetase (γ-GCS) after incubation in different stress media for 96 hours. There was no difference between the control (Ctrl) and patients (*mut^0*) cells under normal conditions. Cells were either grown in normal medium (NM) or were stressed by high-protein (HP) and branched-chain amino acid (IV) load. In *mut^0* cells an increased expression of HO-1 and KEAP-1 could be observed under protein stress and γ-GCS under IV-load. Anserine (2 mM, A) showed its potential antioxidant effect on this condition.

B) Antioxidative capacity, measured by ORAC, was reduced in immortalized proximal tubule epithelial cells (iKTEC) from patients with methylmalonyl-CoA mutase deficiency (*mut^0*) compared to immortalized cells from healthy individuals (Ctrl). Under high-protein conditions (HP) and increased branch-chained amino acids (IV) antioxidative capacity in Mut^0 was only slightly reduced. Co-incubation with anserine (A, 2 mM) at the beginning of the 7-day stress incubation period reduced antioxidant capacity in *mut^0* -cells.

A two-way ANOVA with Šídák's multiple comparison test was used to compare the groups. The mean value with standard deviation is shown. The dots represent the mean value for each patient analyzed in three different experiments.. *=p<0.05, **=p<0.01, ***=p<0.001, ****=p<0.0001. There is no significance between the groups, except the ones indicated in the graphs.

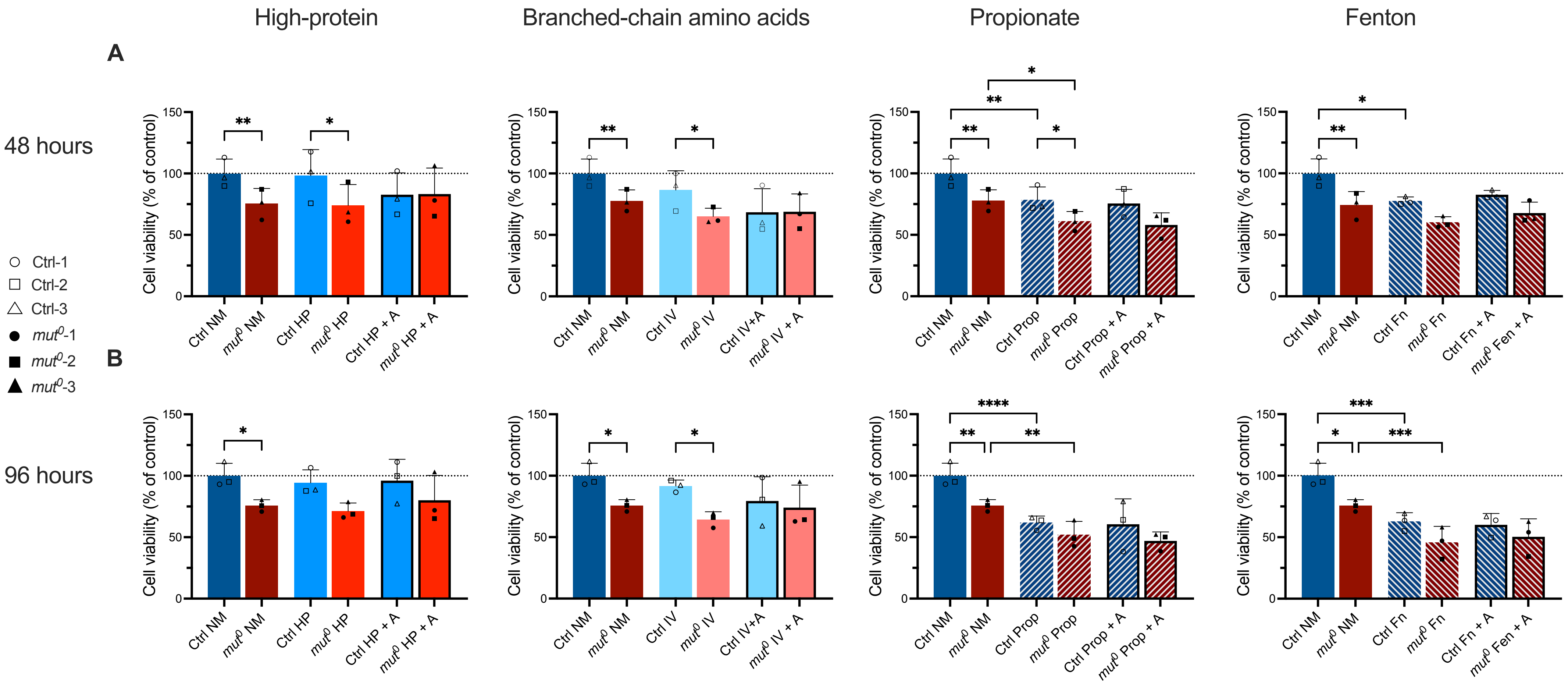

Suppl. Fig. 3: Cell viability of *mut*⁰ cells after 48 and 96 hours of incubation under metabolic stress

Cell viability (A=48 hours, B=96 hours) of iKTEC from *mut*⁰ cells was reduced compared to control cells. Metabolic stress by adding high-protein (HP, 25% FCS), branched-chain amino acids (IV, 1 mM isoleucine, 3 mM valine), propionic acid (Prop, 5 mM) or Fenton (Fen, 3 nM) did not significantly enhance the difference between control and *mut*⁰ cells. Reduced viability by stress was within the same range for control and patient cells. Incubation with anserine did not improve cell viability under any of the conditions. The mean ± standard deviation is shown. Statistical analysis was performed using two-way Anova with Šídák's multiple comparison test. The mean value with standard deviation is shown. The dots represent the mean value for each patient analyzed in three different experiments. *=p<0.05, **=p<0.01, ***=p<0.001, ****=p<0.0001. There is no significance between the groups, except the ones indicated in the graph.
